# A multi-center prospective study of plant-based nutritional support in adult community-based patients at risk of disease-related malnutrition

**DOI:** 10.3389/fnut.2023.1297624

**Published:** 2023-11-10

**Authors:** Marta Delsoglio, Corbin Griffen, Rakshan Syed, Tobias Cookson, Hanorah Saliba, Amanda Vowles, Samuel Davies, Nicola Willey, Jennifer Thomas, Nicola Millen, Nour Odeh, Jayne Longstaff, Naomi Westran, Lindsey Allan, Hannah Offer, Chloe Howell, Meg Sanders, Kirsty Gaffigan, Kirby Garrett, Sally Foster, Agnes Salt, Emily Carter, Sarah Moore, Nick Bergin, Jane Roper, Joe Alvarez, Christine Voss, Teresa Connolly, Clare MacDonald, Tracey Thrower, Darren Sills, Janet Baxter, Rhonda Manning, Lynsey Gray, Karen Voas, Scot Richardson, Anne-Marie Hurren, Daniel Murphy, Susan Blake, Paul McArdle, Sinead Walsh, Lucy Booth, Louise Albrich, Sarah Ashley-Maguire, Joanna Allison, Sarah Brook, Rebecca Capener, Gary P. Hubbard, Rebecca J. Stratton

**Affiliations:** ^1^Research & Innovation, Nutricia Ltd., Trowbridge, United Kingdom; ^2^Preston Hill Surgery, Harrow, United Kingdom; ^3^Trowbridge Health Centre, Trowbridge, United Kingdom; ^4^West Walk Surgery, Yate, United Kingdom; ^5^Cowplain Family Practice, Waterlooville, United Kingdom; ^6^Department of Nutrition and Dietetics, Royal Surrey NHS Foundation Trust, Royal Surrey County Hospital, Guildford, United Kingdom; ^7^Dietetic Department, Thorpe Health Centre, Norfolk Community Health and Care NHS Trust, Norwich, United Kingdom; ^8^Nutrition and Dietetic Department, North Tyneside District General Hospital, Tyne and Wear, United Kingdom; ^9^Department of Nutrition and Dietetics, Airedale General Hospital, Keighley, West Yorkshire, United Kingdom; ^10^Warden Lodge Medical Practice, Cheshunt, United Kingdom; ^11^Rowden Medical Partnership, Chippenham, United Kingdom; ^12^Nutrition and Dietetics, Nottingham University Hospitals NHS Trust, City Hospital Campus, Nottingham, United Kingdom; ^13^Department Nutrition and Dietetics, Kings Cross Hospital, Dundee, United Kingdom; ^14^Dietetics, Victoria Integrated Care Centre, Helensburgh, United Kingdom; ^15^Dietetic Department, Betsi Cadwaladr University Health Board, Denbighshire, United Kingdom; ^16^James Alexander Family Practice, Bransholme South Health Centre, Hull, United Kingdom; ^17^Honiton Surgery, Honiton, United Kingdom; ^18^Birmingham Community Nutrition, Birmingham, United Kingdom; ^19^Yeovil District Hospitals, Yeovil, United Kingdom; ^20^Dietetics, Princess Royal Health Centre, Huddersfield, United Kingdom; ^21^Faculty of Medicine, University of Southampton, Southampton, United Kingdom

**Keywords:** oral nutritional supplement, disease-related malnutrition, plant-based diet, plant-based nutritional support, vegan ONS

## Abstract

**Introduction:**

There is an emerging need for plant-based, vegan options for patients requiring nutritional support.

**Methods:**

Twenty-four adults at risk of malnutrition (age: 59 years (SD 18); Sex: 18 female, 6 male; BMI: 19.0 kg/m^2^ (SD 3.3); multiple diagnoses) requiring plant-based nutritional support participated in a multi-center, prospective study of a (vegan suitable) multi-nutrient, ready-to-drink, oral nutritional supplement (ONS) [1.5 kcal/mL; 300 kcal, 12 g protein/200 mL bottle, mean prescription 275 mL/day (SD 115)] alongside dietary advice for 28 days. Compliance, anthropometry, malnutrition risk, dietary intake, appetite, acceptability, gastrointestinal (GI) tolerance, nutritional goal(s), and safety were assessed.

**Results:**

Patients required a plant-based ONS due to personal preference/variety (33%), religious/cultural reasons (28%), veganism/reduce animal-derived consumption (17%), environmental/sustainability reasons (17%), and health reasons (5%). Compliance was 94% (SD 16). High risk of malnutrition (‘MUST’ score ≥ 2) reduced from 20 to 16 patients (*p* = 0.046). Body weight (+0.6 kg (SD 1.2), *p* = 0.02), BMI (+0.2 kg/m^2^ (SD 0.5), *p* = 0.03), total mean energy (+387 kcal/day (SD 416), *p* < 0.0001) and protein intake (+14 g/day (SD 39), *p* = 0.03), and the number of micronutrients meeting the UK reference nutrient intake (RNI) (7 vs. 14, *p* = 0.008) significantly increased. Appetite (Simplified Nutritional Appetite Questionnaire (SNAQ) score; *p* = 0.13) was maintained. Most GI symptoms were stable throughout the study (*p* > 0.06) with no serious adverse events related.

**Discussion:**

This study highlights that plant-based nutrition support using a vegan-suitable plant-based ONS is highly complied with, improving the nutritional outcomes of patients at risk of malnutrition.

## Introduction

Malnutrition (under nutrition) is often linked to an underlying condition or disease, known as disease-related malnutrition (DRM) (1), and is characterized by inadequate nutritional intakes, increased nutritional requirements, or reduced absorption of nutrients, and arises from a wide range of diseases and/or their treatment (1, 2). In Europe, it is estimated that 33 million people are at risk of becoming malnourished (3), of which ~3 million live in the UK (1, 4). DRM affects individuals across the lifespan; however, it is more common among older individuals as they often have several chronic and progressive co-morbidities (5). Large-scale, multi-center surveys report that DRM risk is widespread in hospitals, care homes, and in the community, and is highly prevalent across many diseases, in particular in patients with cancer, and gastrointestinal and respiratory diseases (6–8).

The consequences of DRM are far-reaching, incorporating a variety of health and social-related manifestations. These include, but are not limited to, weakness, fatigue, loss of independence, depression, impaired quality of life (QoL), delayed recovery from illness, infections, pressure ulcers, and increased morbidity and mortality (2, 9). Patients with DRM also have more general practitioner (GP) visits and inpatient hospital admissions and require longer inpatient hospital stays compared to individuals without DRM (5). Collectively, these adverse outcomes contribute to significant clinical and economic burden on primary and secondary care. In the UK, the overall cost of malnutrition is estimated at £23.5 billion per annum (9), with the health and social care costs of a patient with malnutrition being 3–4 times greater than that of a patient without malnutrition (4). In Europe, the annual cost of DRM is estimated to be €170 billion (3).

For the management of DRM in adults, several nutritional support guidelines have been published by expert groups, including the National Institute for Health and Care Excellence (NICE) (10), the European Society for Clinical Nutrition and Metabolism (ESPEN) (11), the British Association for Parenteral and Enteral Nutrition (BAPEN) (12), and the Malnutrition Pathway (13). These guidelines can be implemented using several nutritional support strategies, including dietary modification, dietetic counseling/advice, and the use of oral nutritional supplements (ONS) (10). Dietary advice acts as the first and most commonly applied approach for patients with the ability to eat; however, a recent meta-analysis in cancer patients concluded that insufficient evidence is available to suggest that dietary advice alone is effective for managing DRM (14). Therefore, patients unable to meet their nutritional requirements through their habitual diet may benefit from the use of ONS alongside food (15).

ONS are energy- and nutrient-dense feeds designed to increase nutritional intake (16). Ready-to-drink, multi-nutrient, liquid ONS, either alone, or in combination with dietary advice, have been shown to be a safe and effective strategy to help patients reach their nutritional requirements and improve patient outcomes (17). These include alleviating disease symptoms, aiding recovery from illness, regaining strength, improving QoL, and reducing occurrence and duration of inpatient hospitalization (2, 10, 15, 18–23). To add, in both hospital and community settings, reviews have highlighted the efficacy of ONS in increasing total energy intake in cystic fibrosis, cardiovascular, chronic obstructive pulmonary disease (COPD), post-surgical, orthopedic, Crohn’s and liver disease, and cancer patients (2, 18, 24). Meta-analyses have also demonstrated increased body weight and handgrip strength alongside reduced clinical complications and hospital readmissions with ONS (25, 26). Economically, evidence suggests that ONS are cost effective relative to dietary advice, with data demonstrating improved quality and quantity of life lived at an acceptable cost (27).

To maximize the clinical and economic benefits related to ONS, it is important that patients achieve good compliance and consume a high percentage (~ ≥ 75%) of what is prescribed (22). Good compliance (78% overall) to ONS in hospital (67%) and particularly community patients (81%) has been reported previously (22); however, compliance can be low (<50%) in some patients and settings, which has been associated with lack of variety and taste fatigue (22, 28, 29). A number of factors have been reported to improve nutritional intake and compliance in patients in need of nutritional support, including palatability, variety, and higher level of ONS choice provided (22, 28, 29). Current evidence therefore suggests that variety and palatability of ONS is key to maximize the clinical and economic effects of ONS.

At present, the majority of ONS available to patients at risk of DRM are based on cow’s milk and contain additional ingredients from animal sources (e.g., vitamin D and fish oils) (30). Problematically, these ingredients may be an issue for patients who do not wish to consume animal-derived products for personal, cultural, religious, animal welfare, environmental, sustainability, intolerance or health reasons, including vegan patients (30–32). Over recent years, there has been a rapidly increasing global interest in reducing animal-derived products and increasing plant-based food consumption (33, 34). Current estimates indicate that ~5% of the European population have adopted a vegetarian eating pattern (35), and 23% are intentionally reducing their intake of animal-derived products (31). In the UK, it is reported that 7.2 million adults follow a plant-based diet, with the number of vegans increasing by 40% (445,428 adults) over the past 12 months (36). While the shift toward plant-based diets is more apparent in younger individuals (37), anecdotal evidence suggests that individuals across the lifespan, including older individuals, are also choosing plant-based alternatives due to generational impacts (38). Indeed, in UK care homes, it has been reported that the number of residents following a vegan diet has risen by 167% since 2014 (39).

The production of animal-derived products is typically environmentally costly (40–43), while overall, it is considered that plant-based food production creates considerably less greenhouse gas (GHG) emissions (44, 45), and a global switch to a more vegetarian diet could reduce not only GHG emissions, but also land and water use (46–48). These findings are significant, as governments (49, 50) and healthcare systems (51) have strategies in place of net zero emissions by 2050. Alongside environmental benefits, the EAT-Lancet Commission summary report on healthy diets from sustainable food systems also highlights that a diet rich in plant-based and fewer animal sources also confers significant improved health benefits in healthy populations (52).

In recognition of the abovementioned beneficial environmental and health effects of increased plant-based food consumption, global organizations, including the Food and Agriculture Organization (FAO) and the World Health Organization (WHO), have published guiding principles for sustainable healthy diets (53). However, while global organizations are calling for, and with rising trends in, increased plant-based food consumption, healthcare professionals (HCPs) currently have limited options for prescribing plant-based ONS to patients who require a plant-based feed. Consequently, the dietary management of DRM in vegan patients and in those who wish to reduce animal-derived consumption is challenging at present. Hence, there is a clear need for plant-based nutritional support options in clinical practice, including ONS. To date, to our knowledge, there are no studies evaluating the use of a plant-based ONS in adult patients. Data on the compliance, tolerance and safety of a plant-based ONS and the effect on nutritional outcomes is needed to build the evidence base to inform clinical practice.

The aim of this pilot study was to evaluate the effects of plant-based nutritional support using a plant-based (vegan suitable) multi-nutrient ONS alongside dietary advice over a 28-day intervention period on compliance (primary outcome), anthropometry, malnutrition risk, dietary intake, appetite, acceptability, gastrointestinal (GI) tolerance, nutritional goal(s), and safety in adult community-based patients at risk of DRM.

## Materials and methods

### Recruitment and study population

Community-based adult patients from 19 primary and secondary healthcare centers in the UK, including National Health Service (NHS) Trusts (*n* = 10), GP surgeries (*n* = 8), and community NHS providers (*n* = 1) were recruited to the study. Patients were screened for the following criteria: (i) ≥16 years of age; (ii) identified as malnourished or at risk of malnutrition using the Malnutrition Universal Screening Tool (‘MUST’) (54), and/or using or requiring ONS at least once daily as part of their nutritional management plan; (iii) expected to receive at least one bottle of the plant-based ONS per day; and (iv) able to provide informed consent. Patients were excluded from the study if they: (i) were receiving parenteral nutrition or ≥ 70% of total energy requirements from enteral tube feeding; (ii) had major hepatic (i.e., decompensated liver disease) or renal dysfunction [requiring filtration or stage 4/5 chronic kidney disease (CKD)]; (iii) had a cancer diagnosis and were receiving systemic anti-cancer therapy (SACT) and likely to experience significant GI disturbances/taste changes due to treatment; (iv) had active/chronic GI disease or impaired GI function; or (v) significant dysphagia/high aspiration risk; (vi) were participating in another clinical intervention study within 1 month of this study; (vii) lacked mental capacity to provide informed consent; (vii) were allergic to any ingredients within the plant-based ONS; and (viii) had an inability/unwillingness to comply with the study requirements. Patients had to complete a minimum of 7 days intervention, as per protocol, to be included in the final analysis.

### Study design and ethics

**Figure 1:**
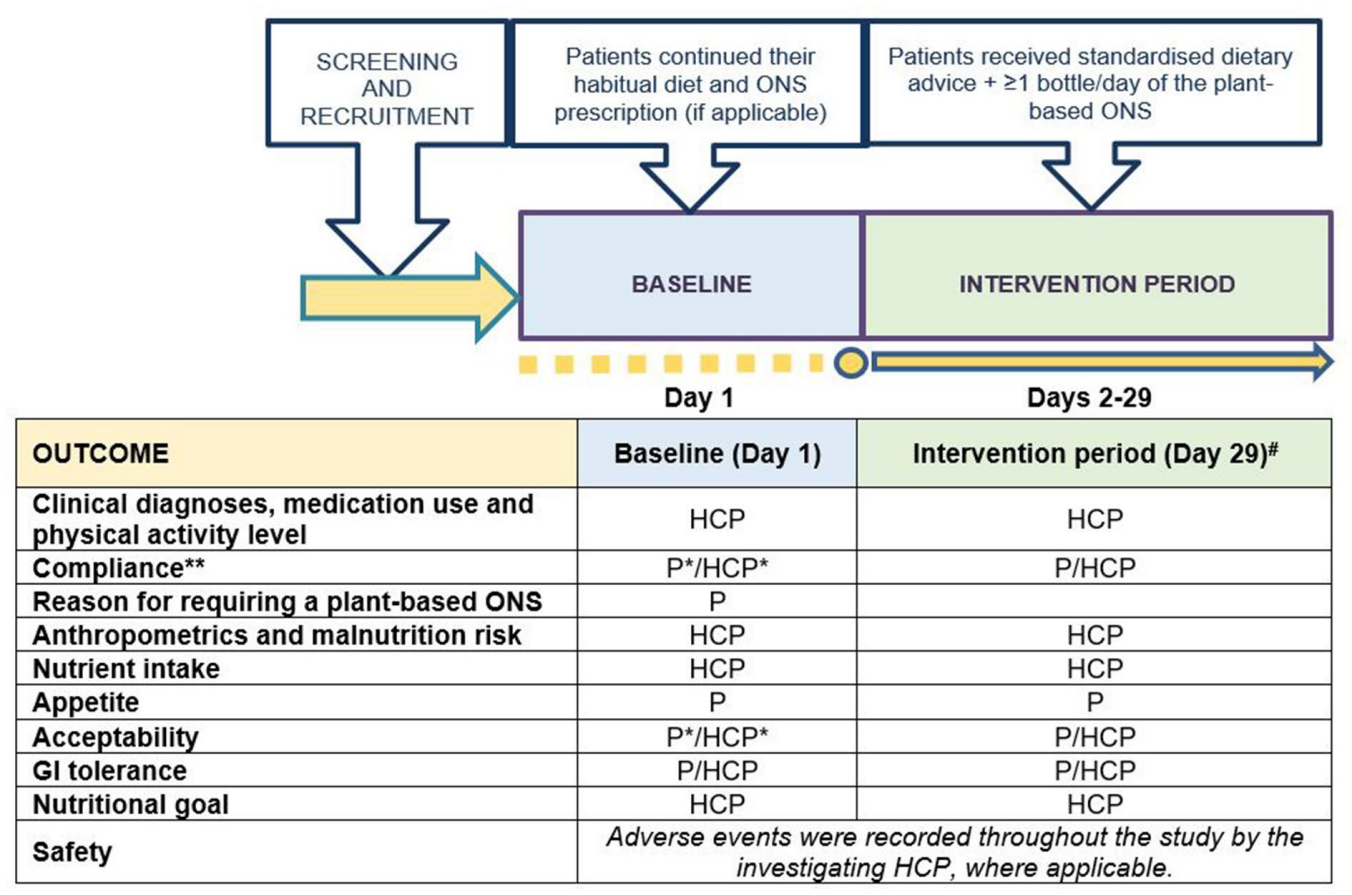
Schematic of the trial design and schedule of assessments. P, outcome recorded by patient/carer; HCP, outcome recorded by patient’s healthcare professional; ^#^, outcomes recorded on Day 29 for all patients unless patient terminated intervention period early, in which case outcomes were recorded on patient’s final intervention day; *, recorded for patients on ONS only; **, recorded every day during the intervention period. Standardized dietary advice was provided via a leaflet by the Malnutrition Pathway (13).

This was a prospective, single-arm, longitudinal, interventional, multi-center pilot study (see Figure 1 for schematic of the trial design and schedule of assessments). During a 1-day baseline, patients continued their habitual diet and ONS prescription as determined by their HCP. Patients then entered the intervention period for 28 days. Outcome measurements were assessed at baseline and at the end of the intervention period. The study protocol was approved by the North West – Greater Manchester West Research Ethics Committee (UK) and was registered at clinicaltrials.gov as part of a larger trial (NCT05257980). UK Health Research Authority (HRA) approval and local NHS R&D/site approval was obtained from all sites involved. The study was conducted in accordance with the Declaration of Helsinki and Good Clinical Practice (GCP) guidelines. All patients provided written informed consent before any study-related procedures were performed.

### Study intervention

Following the 1-day baseline assessment, patients received standardized dietary advice (13) alongside ≥1 bottle/day (to be consumed orally) of a 200 mL ready-to-drink, multi-nutrient, nutritionally complete, 1.5 kcal/mL plant-based (vegan suitable) ONS (Fortisip PlantBased 1.5 kcal, Nutricia Ltd., UK) for 28 days. The standardized dietary advice was provided (via a leaflet) before commencing the intervention period by the investigating HCP, and included advice on eating little and often, snack ideas, and enriching food and nourishing drinks to increase dietary intake (13). The plant-based ONS contained 300 kcal and 12 g of plant-based protein (8.2 g pea and 3.8 g soy protein isolates) per 200 mL bottle (see Table 1 for nutritional composition). All ingredients were derived from non-animal sources, including vitamin D3 from an algal source, deemed to be a viable alternative to animal-derived vitamin D3 with equivalent bioavailability and biological activity (55). The Protein Digestibility Corrected Amino Acid Score (PDCAAS) (56) of the protein (pea and soy blend) within the plant-based ONS was 1.0, which is considered a high quality protein source (57). The appropriate ONS prescription was determined on an individual basis by the investigating HCP responsible for the patient’s care, based on malnutrition risk, clinical judgment, and the patient’s nutritional requirements and preference. Daily timing of consumption of the plant-based ONS was not prescriptive and HCPs informed patients to consume the ONS when convenient. For patients prescribed an ONS at baseline, at least one bottle of their current ONS had to be substituted with one bottle of the plant-based ONS. During the intervention period, all patients had availability of two flavors of the plant-based ONS (Mango Passionfruit and Mocha). Any changes in patients’ ONS prescription, medication use, overall dietary regimen, medical diagnoses and physical activity levels during the intervention period were recorded by HCPs at the end of the study.

**Table 1:** Nutritional composition of the plant-based ONS (Nutricia Ltd).*

	Per 100 mL	Per 200 mL bottle
Energy, kcal	150	300
Carbohydrate, g	18.6	37.2
Carbohydrate, %	49.2	49.2
Protein, g	6.0	12.0
Protein, %	16.0	16.0
Fat, g	5.8	11.6
Fat, %	34.8	34.8
Fiber, g	0.05	0.10

*The plant-based ONS also contained a full range of vitamins and minerals.

### Outcomes

#### Compliance

Compliance (%) with ONS at baseline (if applicable) and with the plant-based ONS was assessed daily throughout the intervention period by each patient recording the amount consumed (mL and bottles/day) compared to the amount prescribed by the investigating HCP. At baseline (if applicable) and at end of intervention, investigating HCPs were asked, using a 5-point Likert scale (strongly agree to strongly disagree), if they were satisfied with the patient’s compliance with the baseline and plant-based ONS, respectively.

#### Reason for requiring a plant-based ONS

At baseline, each patient’s reason for requiring a plant-based ONS was recorded, if applicable. Responses were given by choosing one of the following options: environmental/sustainability reasons, cultural/religious reasons, personal preference/variety, veganism/patient wishes to reduce animal-derived consumption.

#### Anthropometrics and malnutrition risk

Body weight (kg) was measured to the nearest 0.1 kg using a weighing scale, without shoes or heavy clothing, at baseline and end of intervention. Height (m) was measured at baseline only, and with body weight, was used to calculate body mass index (BMI, kg/m^2^) at baseline and end of intervention. The risk of DRM was assessed at baseline and at the end of the intervention period using the Malnutrition Universal Screening Tool (‘MUST’) (54). Malnutrition risk was calculated to be either high risk (‘MUST’ score: ≥2), medium risk (‘MUST’ score: 1), or low risk (‘MUST’ score: 0).

#### Total energy, protein, and micronutrient intakes

Dietary intake of all nutrition provided (including foods, drinks, ONS and enteral tube feeds) was recorded at baseline and at the end of the intervention period via 24 h dietary recall conducted by the investigating HCP. Dietary data were analyzed (inclusive and exclusive of ONS) using nutritional software (Nutritics Academic Edition V5.78, Dublin, Ireland) to calculate total energy (kcal/day), protein (g/day and g/kg/day), vitamin and mineral (mg/day or μg/day, where appropriate) intakes. Actual intakes of energy and protein were compared against the calculated requirements by the investigating HCP using appropriate guidelines (10, 58), where percentage achievements were calculated. Intakes of vitamins and minerals were also calculated as a percentage of UK age- and sex-specific reference nutrient intake (RNI) values (59), where applicable.

#### Appetite

Appetite profile was assessed by the patient in the morning (fasted) at baseline and at the end of the intervention period using the Simplified Nutritional Appetite Questionnaire (SNAQ) (60), a 4-item questionnaire which assesses taste, appetite, fullness and frequency of eating. Each question was scored between 0–5, with a total score between 0–20 (a higher score indicates increased appetite).

#### Acceptability

Acceptability of the baseline ONS (if applicable) and of the plant-based ONS was assessed by each patient at baseline and at the end of the intervention period, respectively, using a standardized questionnaire on a 7-point Likert scale (strongly agree to strongly disagree). Questions related to ease and quickness of consumption, volume, convenience, how well the ONS fitted in the patient’s diet, ease of opening, bottle quality/durability, consistency, smell, appearance, taste, aftertaste and overall likeability. Patients were also asked to rate the taste, aftertaste, smell, appearance and thickness of their baseline ONS (if applicable) and of the plant-based ONS out of 10 (1 = very poor and 10 = excellent). At baseline (if applicable) and at the end of the intervention period, investigators were asked on a 5-point Likert scale (strongly agree to strongly disagree) if they were satisfied with the patient’s ONS acceptability.

#### Gastrointestinal tolerance

The incidence and intensity of GI symptoms (diarrhea, constipation, nausea, vomiting, abdominal pain, bloating, flatulence and burping) were recorded by each patient on a 4-point Likert scale (absent, mild, moderate, severe) at baseline and at the end of the intervention period. Stool appearance was recorded at baseline and at the end of the intervention period using the Bristol Stool Chart (61). Patients and investigators also rated how well their baseline ONS (if applicable) and the plant-based ONS were tolerated on a 7-point and 5-point Likert scales (strongly agree to strongly disagree), respectively.

#### Nutritional goal

Nutritional goal(s) related to the introduction of the plant-based ONS over the intervention period were set for each patient by investigating HCPs at baseline. These could relate to weight gain, compliance, increased nutritional intake, GI tolerance, or any other relevant outcome. Achievement of these goal(s) were recorded at the end of the intervention period.

#### Safety

Adverse and serious adverse events (SAEs) were recorded throughout the study, where information concerning the intensity and potential relatedness to the plant-based ONS were determined.

### Statistical analysis

An *a priori* power calculation was conducted based on compliance to ONS (the primary outcome measure) reported in community-based patients (81 ± 13%) (22). Based on an assumed SD of 13% compliance throughout the intervention period, power of 80% and an alpha of 0.05, a sample size of 19 patients was required to detect 90% compliance (9% increase over time). Therefore, a target sample size of 25 patients, allowing for a dropout rate of ~25%, was deemed reasonable for this pilot study. Patients completing a minimum of 7 days of the intervention were included in final analysis using SPSS v27 (IBM corp., New York, United States). Data were checked for normality using the Shapiro–Wilk test. For continuous data, paired samples *t*-tests were used for comparisons of two time points (baseline vs. end of the intervention). One-way ANOVA or repeated-measures ANOVA were used for sub-analyses, where applicable (e.g., comparison of dose, timing of consumption). For non-parametric data, the Wilcoxon signed-rank test was used. Descriptive data (means, percentages, standard deviations) are provided, where applicable. Categorical data were analyzed using appropriate methods (e.g., chi-squared). Significance was assumed at a level of *p* < 0.05.

## Results

### Recruitment and patient characteristics

**Figure 2:**
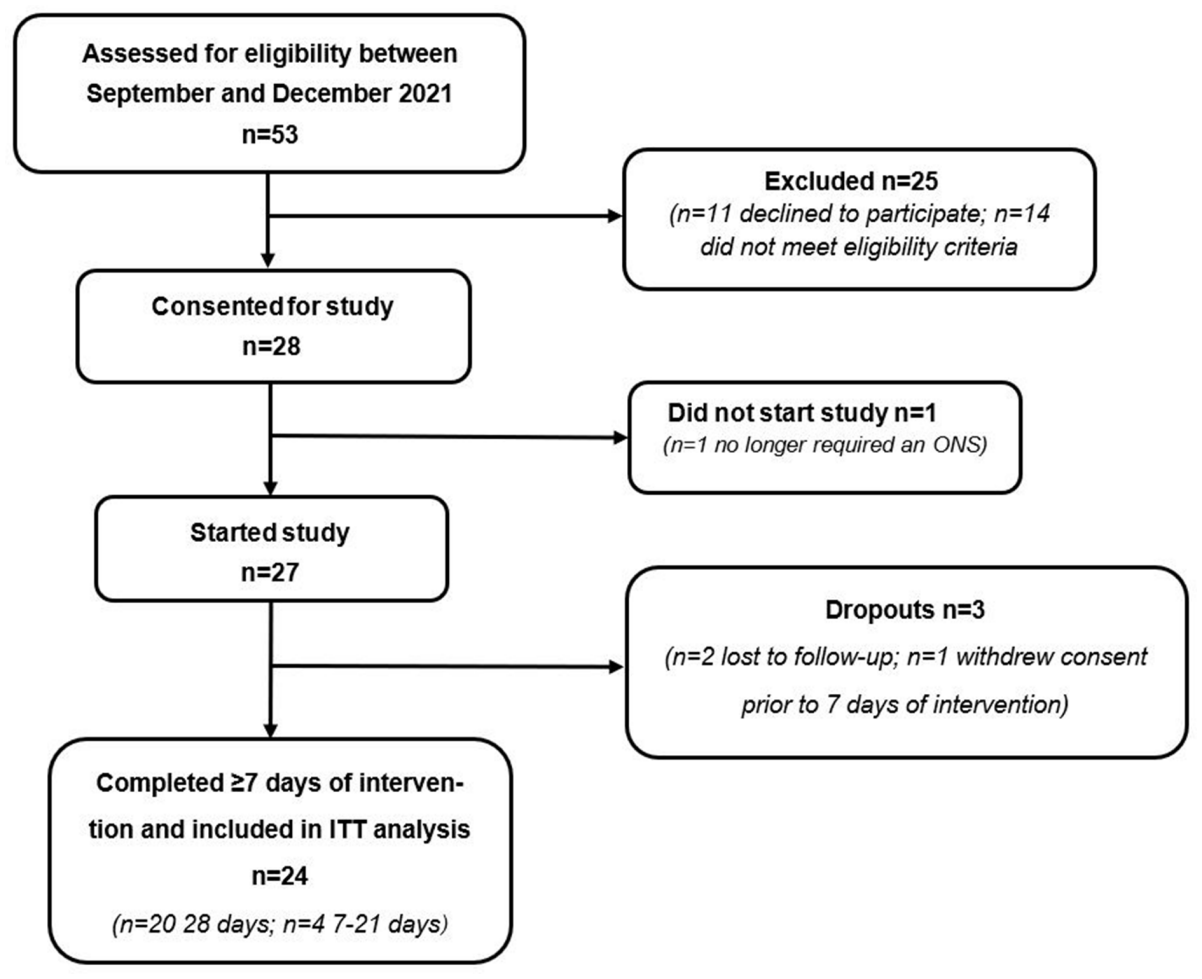
Flow chart of patient recruitment and study participation. ITT, intention-to-treat.

A total of 53 patients were assessed for eligibility to participate, of which *n* = 28 were deemed eligible and consented to take part (see Figure 2 for participant flow). Due to significant clinical improvement, *n* = 1 no longer required an ONS and consequently did not start the study. Of the 27 patients that completed baseline measures, *n* = 1 withdrew prior to day 7 and *n* = 2 were lost to follow-up. Therefore, 24 patients completed a minimum of 7 days intervention as per protocol and were included in the final analysis. Of the 24 patients who completed the study, 83% (*n* = 20) were recruited from primary care sites (GP surgeries) and 17% (*n* = 4) were recruited from secondary care sites (NHS trusts).

Patients’ ages ranged from 29 to 84 years (mean = 59, SD 18) with 18 patients identified as females, and 6 as males. Patients had a baseline BMI of 19.0 kg/m^2^ (SD 3.3) and presented with multiple primary diagnoses (see Table 2 for mean patient baseline characteristics, and Supplementary Table S1 for individual patient characteristics). Medications prescribed to patients included anti-hypertensives (*n* = 7), anti-diabetic medication (*n* = 7), anti-depressants (*n* = 6), statins (*n* = 5), protein pump inhibitors (*n* = 5), bronchodilators (*n* = 4), thyroxine (*n* = 3), painkillers (*n* = 3), anticholinergics (*n* = 2), oral contraceptives (*n* = 2), bisphosphonates (*n* = 2), cholecalciferol (*n* = 2), and laxatives, corticosteroids, monoclonal antibodies, chemotherapy agents, anti-epileptic and anti-mobility medication, and amino salicylates (*n* = 1 for all). Most patients (*n* = 23) resided in their own/family home and 1 patient resided in sheltered accommodation. Eighteen patients were either retired (*n* = 5) or not working (*n* = 13), and 6 patients were employed either full- (*n* = 4) or part-time (*n* = 2). Overall, patients had low physical activity levels: *n* = 2 were bed rest, chair or bed bound, *n* = 8 were very sedentary with little activity, *n* = 8 performed light activity, and *n* = 6 performed moderate activity. Twenty patients had a ‘MUST’ score of 2 (high risk of malnutrition), 3 patients had a ‘MUST’ score of 1 (medium risk of malnutrition), and 1 patient had a ‘MUST’ score of 0 (low risk of malnutrition) but was already prescribed an ONS alongside dietary advice prior to entering the study due to difficulty in consuming food and potential for continuing weight loss. A total of 13 patients were following dietary advice alone prior to the study but required an ONS to further increase energy intake and body weight, and 8 patients were already prescribed an ONS. All patients were 100% orally fed with no patients receiving enteral tube feeding.

**Table 2:** Baseline patient characteristics (*n* = 24).

	Mean (SD)
Age, years	59 (18)
Height, cm	162 (9)
Weight, kg	49.9 (9.9)
BMI, kg/m^2^	19.0 (3.3)
Energy requirements, kcal/day	1516 (467)
Energy intake, kcal/day	1189 (532)
Protein requirements, g/day	59.2 (19.7)
Protein intake, kcal/day	44 (21)
Protein intake, g/kg/day	0.9 (0.4)

	*n* (%)
Female	18 (75)
Male	6 (25)
Following dietary advice	13 (54)
Prescribed ONS	8 (33)
‘MUST’ score (risk classification)	
2 or more (High risk)	20 (83)
1 (Medium risk)	3 (13)
0 (Low risk)	1 (4)
Primary diagnosis	
CVD	6 (25)
Cancer	5 (21)
COPD	4 (16)
Anorexia	2 (7)
Anxiety	2 (7)
Epilepsy	1 (4)
Endometriosis	1 (4)
Rheumatoid arthritis	1 (4)
Thyroid disorder	1 (4)
Ulcerative colitis	1 (4)

BMI, body mass index; COPD, chronic obstructive pulmonary disease; CVD, cardiovascular disease; ‘MUST’, Malnutrition Universal Screening Tool; ONS, oral nutritional supplement.

The plant-based ONS was prescribed by investigating HCPs to meet patients’ needs due to low BMI (*n* = 15), weight loss (*n* = 4), unable to maintain/gain weight (*n* = 4), and due to inability to meet nutritional requirements with dietary advice alone (*n* = 1). One patient was prescribed 3 bottles/day (600 mL), 7 patients were prescribed 2 bottles/day (400 mL), and 16 patients were prescribed 1 bottle/day (200 mL). Mean prescription was 275 mL/day (SD 115) providing 412 kcal/day (SD 172) and 17 g/day (SD 7) of protein. Fifteen patients (63%) were prescribed Mango Passionfruit flavor only, 2 patients (8%) Mocha flavor only, and 7 patients (29%) were prescribed both flavors. During the intervention period, patients consumed the plant-based ONS for 26 days (SD 6), with most patients (*n* = 20) taking the plant-based ONS for the full 28-day intervention period. Four patients consumed the plant-based ONS for between 7–21 days and did not complete the full 28-day intervention period due to no longer wanting to take part in the study (*n* = 1), and onset of mild–moderate GI symptoms (*n* = 3, detailed later). Most patients (*n* = 14, 58%) consumed the plant-based ONS between meals either in the morning or afternoon, however some consumed the ONS with breakfast (*n* = 3, 13%), lunch (*n* = 4, 16%), or dinner (*n* = 3, 13%). The plant-based ONS prescription did not change during the intervention period for any patients. No changes in clinical conditions, physical activity or use of medications were recorded during the intervention period.

### Compliance

Compliance with the plant-based ONS was excellent, with a mean compliance of 94% (SD 16) versus HCP prescription. Compliance did not differ between prescribed volume (200 mL: 97% (SD 9); 400 mL: 87% (SD 28); 600 mL (*n* = 1): 96%, *p* = 0.47) or flavors (Mango Passionfruit: 96% (SD 9); Mocha: 100% (SD 0); both: 87% (SD 28), *p* = 0.39). In patients already prescribed an ONS at baseline (*n* = 8), compliance increased by 25% (SD 63) with the plant-based ONS, from 63% (SD 52) at baseline to 88% (SD 26) at end of intervention, however the difference was not statistically significant (*p* = 0.29). Most investigating HCPs (83%) strongly agreed or agreed that they were satisfied with their patient’s compliance with the plant-based ONS, which was significantly greater compared to baseline ONS (75%, *p* = 0.03).

### Reason for requiring the plant-based ONS

**Figure 3:**
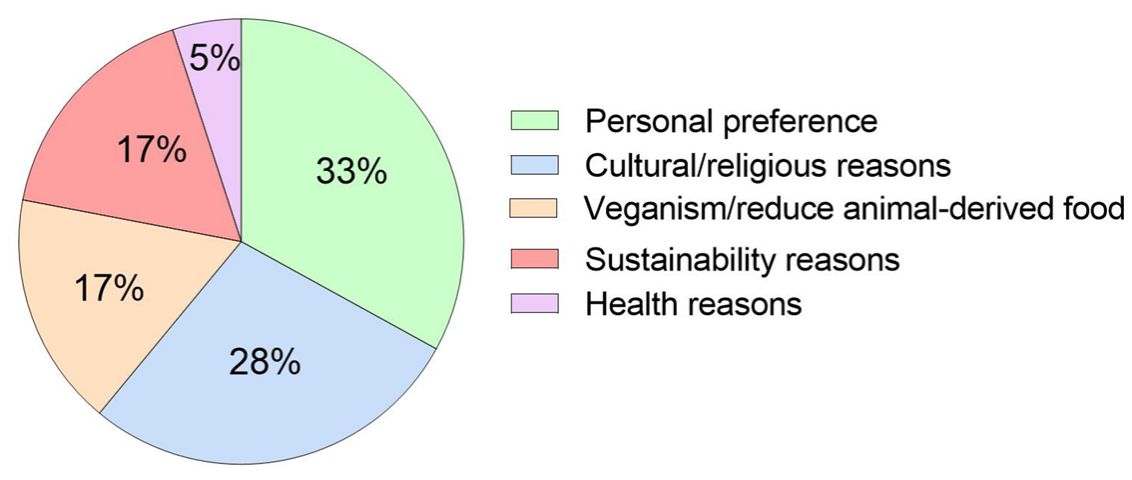
Reasons (%) why patients at risk of DRM required a plant-based ONS (*n* = 18).

The ONS was required or preferred to be plant-based in 75% (*n* = 18) of patients, which was significantly greater than the 25% (*n* = 6) of patients who did not specifically require the ONS to be plant-based (*p* = 0.01). Patients (*n* = 18) who required a plant-based ONS did so due to personal preference (*n* = 6), religious/cultural reasons (*n* = 5), veganism/wish to reduce animal-derived consumption (*n* = 3), environmental/sustainability reasons (*n* = 3), and health reasons (*n* = 1), with no significant difference observed between reasons (*p* = 0.25, Figure 3).

### Anthropometrics and malnutrition risk

**Figure 4:**
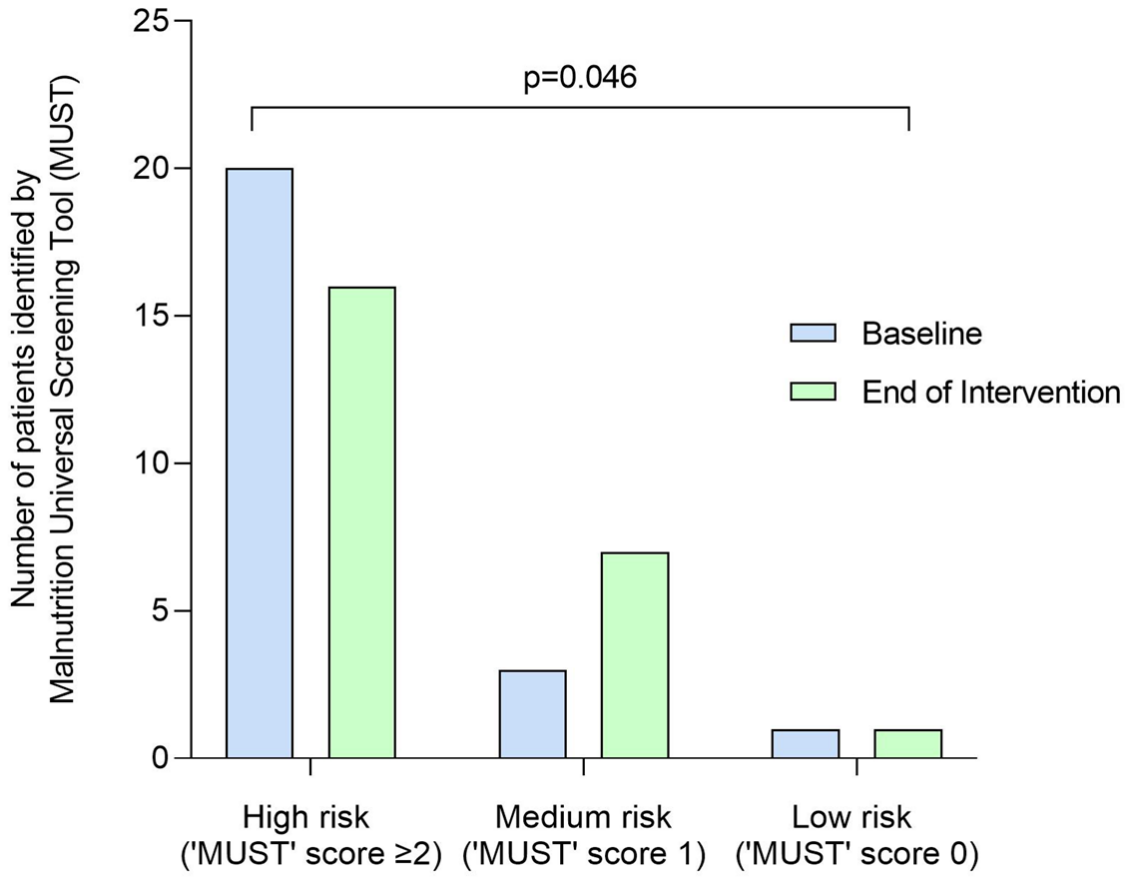
Number (*n*) of patients identified as at high risk (‘MUST’ score = ≥2), medium risk (‘MUST’ score = 1) and low risk (‘MUST’ score = 0) of malnutrition at baseline and end of intervention by the Malnutrition Universal Screening Tool (MUST). Data were analyzed by Wilcoxon signed-rank test.

Body weight significantly increased from baseline to end of intervention by +0.6 kg (SD 1.2, *p* = 0.02), from 50.1 kg (SD 10.0) to 50.7 kg (SD 10.1), with BMI significantly increasing by +0.2 kg/m^2^ (SD 0.5, *p* = 0.03), from 19.0 kg/m^2^ (SD 3.4) to 19.2 kg/m^2^ (SD 3.4). ‘MUST’ score significantly improved at end of intervention with the plant-based ONS, with 20 patients classified as at high risk of malnutrition (‘MUST’ score ≥ 2) at baseline, reducing to 16 patients at end of intervention (*p* = 0.046, Figure 4).

### Total energy, protein, and micronutrient intakes

**Figure 5:**
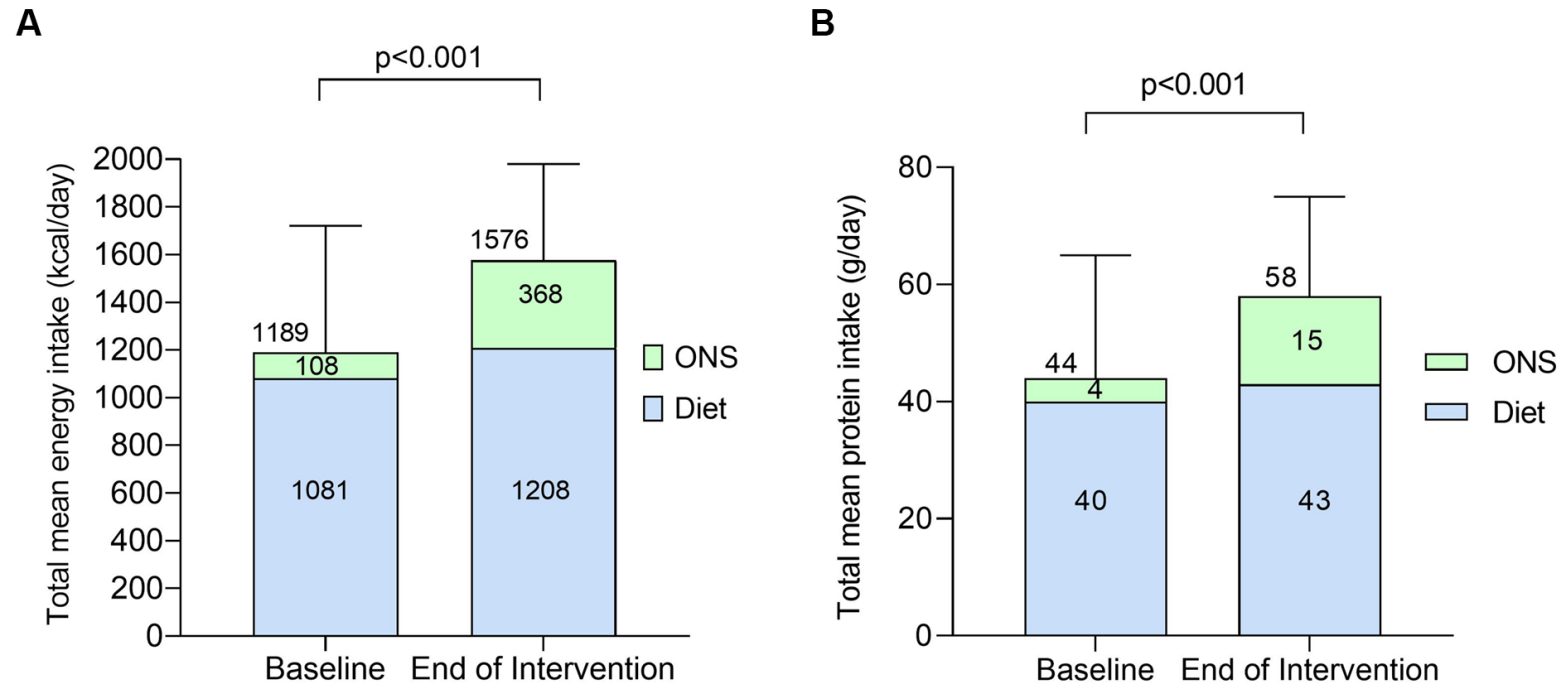
Total mean energy [**(A)**, kcal/day] and protein intake [**(B)**, g/day] from diet alone and with ONS at baseline and end of intervention [*n* = 23, means (SD)]. Data were analyzed by paired samples *t*-test.

Total mean energy intake increased significantly by +387 kcal/day (SD 416, *p* < 0.0001, Figure 5A) from baseline to the end of intervention with the plant-based ONS. The increase in total mean energy intake was predominantly driven by an increase in mean energy intake from ONS (accounting for 67% of the increase), which significantly increased by +260 kcal/day (SD 209, *p* < 0.001) from baseline to the end of intervention. Mean energy intake from diet alone (without baseline and the plant-based ONS) also increased (accounting for 33% of the increase in total energy intake) by +126 kcal/day (SD 352) from baseline to the end of intervention, however the increase was not statistically significant (*p* = 0.08). Total mean energy intake as a percentage of mean estimated requirements increased significantly from 80% (SD 26) at baseline to 111% (SD 29) at the end of intervention with the plant-based ONS (*p* < 0.001).

Total mean protein intake increased significantly by +14 g/day (SD 39, *p* = 0.03, Figure 5B) from baseline to the end of intervention with the plant-based ONS. Relative to body weight, mean protein intake also significantly increased by +0.3 g/kg/day (SD 0.6, *p* = 0.02) from baseline [0.9 g/kg/day (SD 0.5)] to the end of intervention [1.2 g/kg/day (SD 0.5)]. The increase in total mean protein intake was driven by an increase in mean protein intake from ONS (accounting for 79% of the increase), which significantly increased by +11 g/day (SD 10, *p* < 0.001) from baseline to the end of intervention. Mean protein intake from diet alone (without baseline and the plant-based ONS) remained stable during the study (*p* = 0.38). Total mean protein intake as a percentage of estimated requirements increased significantly from 76% (SD 32) at baseline to 106% (SD 43) at the end of intervention with the plant-based ONS (*p* = 0.009).

Mean total micronutrient intakes were significantly greater at the end of intervention with the plant-based ONS compared to baseline for potassium, calcium, iron, copper, zinc, selenium, vitamin D, and vitamin C (*p* < 0.05, Table 3). All other mean micronutrient intakes were either maintained or increased, but not significantly, from baseline to the end of intervention (*p* ≥ 0.06). At baseline, there were 7/20 micronutrients for which the mean total daily intake met the UK RNI, which increased to 14/20 micronutrients at the end of intervention with the plant-based ONS (*p* = 0.008).

**Table 3:** Total micronutrient intakes and % of UK reference nutrient intake (RNI) at baseline and end of intervention [mean (SD)].

	Baseline	End of Intervention	% of UK RNI (59)		*p* value
			Baseline	End of Intervention	
Minerals					
Sodium (mg/day)	1430 (781)	1620 (823)	89 (49)	101 (51)	0.38
Potassium (mg/day)	1538 (884)	2385 (896)	44 (25)	68 (25)	<0.001*
Chloride (mg/day)	2373 (1252)	2538 (1168)	95 (50)	102 (47)	0.62
Calcium (mg/day)	521 (394)	835 (304)	74 (56)	119 (43)	0.002*
Phosphorus (mg/day)	759 (559)	945 (296)	138 (102)	172 (54)	0.13
Magnesium (mg/day)	173 (106)	212 (68)	61 (36)	76 (24)	0.06
Iron (mg/day)	7.7 (4.8)	11.0 (3.6)	78 (49)	116 (41)	0.002*
Copper (mg/day)	0.7 (0.5)	1.2 (0.4)	59 (38)	103 (36)	<0.001*
Zinc (mg/day)	5.2 (3.5)	8.4 (2.6)	68 (44)	111 (33)	0.001*
Manganese (mg)	2.0 (1.1)	2.4 (1.0)	NA	NA	0.22
Iodine (μg/day)	81 (95)	114 (74)	58 (68)	82 (53)	0.21
Selenium (μg/day)	25 (26)	42 (14)	38 (37)	65 (20)	0.01*
Vitamins					
Vitamin A (μg RE/day)	607 (606)	823 (718)	94 (91)	132 (119)	0.28
Vitamin D (μg/day)	4.1 (8.5)	8.1 (3.1)	42 (84)	79 (31)	0.047*
Vitamin E (mg/day)	8.6 (9.2)	12.2 (5.4)	NA	NA	0.10
Vitamin C (mg/day)	54 (80)	103 (167)	135 (199)	257 (417)	0.046*
Vitamin K (μg/day)	29 (54)	31 (16)	NA	NA	0.76
Thiamin (mg/day)	1.3 (1.4)	1.3 (0.5)	136 (148)	147 (57)	0.92
Riboflavin (mg/day)	1.2 (1.4)	1.4 (0.6)	103 (115)	117 (52)	0.68
Niacin (mg NE/day)	18.9 (15.5)	20.9 (9.7)	136 (101)	155 (80)	0.65
Vitamin B6 (mg/day)	1.4 (1.9)	1.4 (0.4)	112 (143)	110 (30)	0.87
Folate (μg DFE/day)	144 (102)	155 (73)	72 (51)	78 (37)	0.74
Vitamin B12 (μg/day)	3.3 (4.1)	3.3 (2.4)	220 (276)	223 (157)	0.99
Biotin (μg/day)	25 (25)	37 (15)	NA	NA	0.06
Pantothenic acid (mg/day)	4.2 (5.5)	5.5 (1.8)	NA	NA	0.30

*Significant *p* value (<0.05). DFE, dietary folate equivalents; NA, not applicable; NE, niacin equivalents; RE, retinol equivalents.

### Appetite

SNAQ score was maintained at end of intervention with the plant-based ONS [11.8 (SD 3.5)] compared to baseline [11.3 (SD 3.0), *p* = 0.13]. SNAQ score did not differ between the dose of plant-based ONS prescribed (*p* = 0.52) or time of day consumed (*p* = 0.72).

### Acceptability

**Figure 6:**
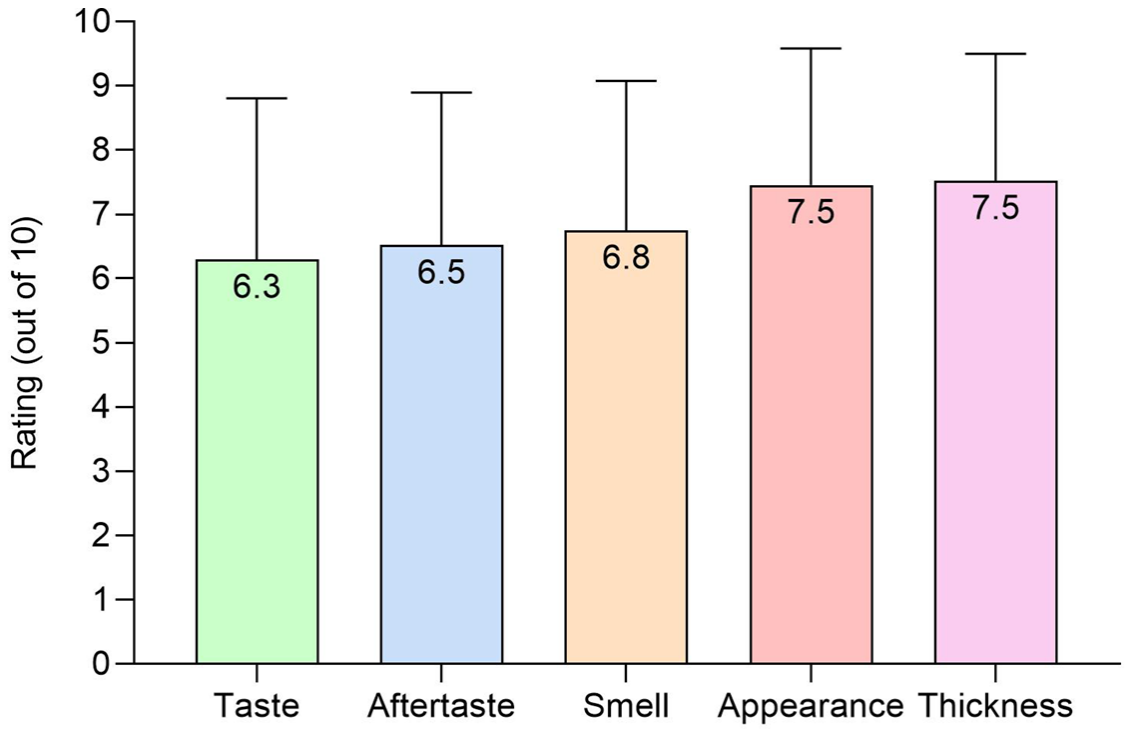
Sensory outcomes (out of 10) for the plant-based ONS at end of intervention [*n* = 24, means (SD)].

Patients rated the plant-based ONS as good to excellent (mean score ≥ 6.3 out of 10) for taste, aftertaste, smell, appearance, and thickness (Figure 6). In patients (*n* = 8) prescribed an ONS at baseline, no significant differences occurred for any sensory outcome between baseline and the plant-based ONS (*p* > 0.06).

The plant-based ONS was highly accepted by patients, with most strongly agreeing or agreeing that the ONS was convenient (92%), easy to drink (83%), fitted in with their diet (83%), and likeable overall (70%). Further acceptability outcomes were also rated positively (≥67% strongly agree or agree responses), as can be seen in Table 4. In patients (*n* = 8) already prescribed an ONS at baseline, no significant differences in acceptability outcomes were observed between baseline and the plant-based ONS (*p* > 0.13). Most investigating HCPs (88%) strongly agreed or agreed that they were satisfied with their patient’s acceptability with the plant-based ONS, which was the same as that at baseline for those patients taking an ONS (88%, *p* = 0.16).

**Table 4:** Acceptability outcomes for the plant-based ONS at end of intervention.

Acceptability outcomes	Strongly agree or agree responses (%)
Easy to drink	83
Adequate volume	88
Convenience	92
Fits in with my current diet	83
Well tolerated	79
Easy to open	83
Good quality/durable (the bottle)	100
Quick for me to drink	75
Adequate consistency	67
Pleasant to smell	67
Appealing to look at (the liquid)	71
Enjoyable to taste	67
Pleasant aftertaste	70
Likeable overall	70

### Gastrointestinal tolerance

Most gastrointestinal symptoms were absent (74–96% of patients) at baseline with a few occurrences (4–22% of patients) of mild–moderate symptoms reported for each GI symptom. At the end of intervention while taking the plant-based ONS, diarrhea, constipation, nausea, vomiting, abdominal discomfort/pain, and bloating remained stable (*p* > 0.06); however, mild symptoms of flatulence (17% vs. 36%, *p* = 0.001) and burping (4% vs. 23%, *p* = 0.046) increased. These symptoms may have been due to the addition of an ONS into the diet of patients new to ONS, as no significant differences in any GI symptoms occurred between baseline and the end of intervention in patients already taking an ONS at baseline (*p* > 0.06, *n* = 8). HCPs were not concerned with these increased symptoms, with 88% agreeing or strongly agreeing that they were satisfied with their patient’s tolerance with the plant-based ONS, compared to 63% at baseline (*p* = 0.12). Most patients (79%) also strongly agreed or agreed that they tolerated the plant-based ONS well, similar to that of patients (*n* = 8) taking an ONS at baseline (71%, *p* = 0.18). No change in stool appearance occurred between baseline and end of intervention (*p* = 0.13).

### Nutritional goal

At baseline, 67% (*n* = 16) of patients’ nutritional goal set by the investigating HCPs was weight gain, 29% (*n* = 7) was weight maintenance, and *n* = 1 patient’s nutritional goal was maintenance of energy levels. At end of intervention, 67% of patients (*n* = 16) met their nutritional goal, including *n* = 6 (86%) achieving their weight maintenance goal.

### Safety

There were no major safety concerns relating to the plant-based ONS reported during the intervention period. No SAEs were recorded. Individual symptoms of mild–moderate abdominal pain, acid reflux and nausea were reported in *n* = 3, which were recorded as possibly related to the plant-based ONS. One patient experienced a urinary tract infection, but the patient’s investigating HCP deemed this unrelated to the plant-based ONS.

## Discussion

To our knowledge, the present, single-arm, longitudinal, interventional, multi-center pilot study is the first to investigate and provide novel data on the value of plant-based nutritional support using a ready-to-drink, nutritionally complete, plant-based (vegan suitable) ONS alongside dietary advice in adult community-based patients at risk of DRM. Overall, this trial shows for the first time that a ready-to-drink plant-based ONS is highly complied with, tolerated, acceptable and safe, and is effective at increasing total energy, protein and micronutrient intakes, body weight and BMI, and reducing malnutrition risk. The trial also confirms that there are a variety of reasons why patients at risk of DRM may require or prefer a ready-to-drink, plant-based ONS.

Previous studies have demonstrated that ONS, either alone, or in combination with dietary advice, are a safe and effective intervention to help patients achieve their nutritional requirements and improve patient outcomes (2, 10, 15, 18–23, 25–27). However, for ONS to be clinically and cost effective, it is important that patients achieve good compliance (i.e., ≥75%) (22). In the present study, we observed excellent compliance (94%) with the plant-based ONS, which was higher than the compliance level of 78% reported in a systematic review of studies across a diverse range of patient groups in hospital and community settings assessing a variety of ONS (22). Our compliance data is in line with findings from Liljeberg et al. (28), who reported high compliance (93%) with ONS in patients at risk of DRM measured by frequency questionnaire. Regrettably, comparison of compliance data with other ready-to-drink plant-based ONS is challenging due to a scarcity of ready-to-drink plant-based ONS available to patients.

The excellent compliance with the plant-based ONS was likely attributed to the high acceptability of the ONS, which is fundamental and can be affected by product (e.g., ONS palatability, volume, thickness, flavor) and personal factors (e.g., attitudes and motives, age and sensory decline) (62, 63). Regarding sensory outcomes, patients scored all outcomes >6/10 for the plant-based ONS, which translates to a positive score for ONS acceptability (64). Most patients (>70%) also strongly agreed or agreed that the plant-based ONS was convenient, easy to drink, and likeable overall, which was similar for patients (*n* = 8) prescribed an ONS at baseline. These findings concur with those presented in a systematic review of acceptability of consumers to plant-based protein sources (65). Concerning personal factors, particularly attitudes and motives, 75% of patients specifically required or preferred to be prescribed a plant-based ONS, which broadly aligns with the 83% of patients who strongly agreed or agreed that the plant-based ONS fitted in well with their current diet. This suggests that the plant-based nature of the ONS was a significant contributing factor to ONS acceptability in most patients in this study, even though requirement for a plant-based ONS was not part of the study inclusion criteria. This data is not surprising, especially considering that over recent years, there has been a rapidly increasing global interest in reducing animal-derived products and increasing plant-based food consumption (31, 32). While anecdotal data suggests that the shift toward plant-based diets is more apparent in younger individuals (37), individuals across the lifespan, including older individuals and patients, are also opting for plant-based alternatives (38, 39, 66). In agreement, while the mean age of patients at risk of DRM in the present study was younger (59 years, SD 18, range 29–84 years) than that of previous studies evaluating ONS (79–86 years) (67–71), a significant proportion of patients (*n* = 9, 38%) were > 70 years. Data from this study therefore confirms that plant-based ONS are required for patients across the adult lifespan. Furthermore, while 25% of patients did not specifically require a plant-based ONS, data from this study shows that a plant-based ONS is still a viable and effective alternative ONS in these patients.

Reasons for a requiring a plant-based ONS by patients in this study were varied, and included personal preference/variety, religious/cultural reasons, veganism/wish to reduce animal-derived consumption, environmental sustainability and health reasons. These motivations were similar to that previously reported at a population level (32) and accentuate the multifaceted need for plant-based ONS in clinical practice. Interestingly, a systematic review highlighted that variety is a key factor to aid ONS compliance (22); therefore, the percentage of patients requiring a plant-based ONS due to personal preference/variety in this study is highly likely to aid compliance in these patient groups.

The plant-based ONS elicited significant nutritional improvements in as little as ~26 days, with a magnitude similar to that of other studies evaluating the clinical effectiveness of ONS, most notably milk-based ONS (15, 72, 73). This was likely due to the suitable plant-based ONS nutritional profile, that provided additional energy and protein intake. A meta-analysis reported a significant increase in energy intake (+423 kcal/day) in 1,414 cancer patients with DRM (72). More recently, a randomized trial in older malnourished people in primary care demonstrated that ONS + dietary advice increased energy (+401 kcal/day) and protein intake (+15 g/day), body weight (+0.8 kg), and reduced malnutrition risk greater than dietary advice alone (15). Also, while data on the clinical effectiveness of plant-based ONS is limited, data from this study is consistent with a recently published case study, which showed that a plant-based ONS promoted weight gain in a burn patient (74).

At present, there are anecdotal concerns among HCPs that switching animal-derived protein consumption for plant-based alternatives may lead to reduced protein quality, with potential adverse effects on patient outcomes (75). However, there are currently no recommendations for protein quality of ONS for the treatment of DRM in clinical practice. Nevertheless, where possible, recommendations set for healthy populations for essential amino acid (EAA) requirements, e.g., the FAO and WHO (76, 77), should be followed to ensure the metabolic demands of patients are met. While the PDCAAS is typically lower for plant-based compared to animal-derived protein sources (40), pea and soy protein isolates have high digestibility (>95%), similar to that of dairy proteins (78–83). Previous studies have also shown that plant-based diets are effective at improving functional outcomes (84) and reducing the risk of developing frailty (85), and that there are no differences in lean mass or muscle strength outcomes between plant-based and animal protein sources when protein intake is sufficient (40, 86–88). The protein source of the plant-based ONS used in this study (containing 32% soy and 68% pea protein isolates) is sufficient to meet the minimum EAA requirements for adults (76, 77) and has a PDCAAS of 1.0, therefore considered high quality (57). Further, as previously highlighted, the plant-based ONS elicited significant nutritional benefits, increasing relative protein intake to 1.2 g/kg/day, meeting the recommended protein requirements for these patient groups (89, 90). Therefore, while measures of lean mass and functional outcomes were not obtained in this study, data from the present and aforementioned studies imply that plant-based ONS will not adversely impact and will more likely improve patient outcomes long-term.

An important outcome of the present study was that the plant-based ONS, which is nutritionally complete with a full range of vitamins and minerals, maintained or increased micronutrient intakes. Notably, significant increases in mean intakes of calcium, iron, zinc and vitamin D were observed, and the number of micronutrients meeting the UK age- and sex-specific RNI (59) increased. These finding are significant, as previous studies have reported micronutrient deficiency, particularly deficiency of vitamin B12, iron, calcium, vitamin D, iodine and zinc, in individuals following a plant-based or vegan diet (91). Micronutrient deficiency may negatively affect body function, mainly the nervous, skeletal and immune systems, and has also been related to hematological disorders (91). As such, potential related deficiencies in patients following a plant-based or vegan diet is a concern for HCPs. Nevertheless, the results of this study demonstrate that patients who require an ONS and wish to follow a plant-based diet, or consume a plant-based ONS, can do so without any adverse effects on micronutrient intakes.

It is well-known that aging (92) and disease (93) can affect appetite. Therefore, an important outcome of this study was maintenance of patients’ appetite while taking the plant-based ONS. In fact, energy intake from diet alone increased, albeit non-significantly, by 126 kcal/day during the intervention period. Our data is in agreement with previous studies that have established that ONS maintains perceived appetite and do not significantly adversely affect energy intake from diet alone in patients at risk of DRM, hence resulting in an increase in overall energy intake due to ONS (25, 94–96). While data on the effects of plant-based protein sources on appetite in malnourished patients is scarce, current evidence points toward the hypothesis that plant-based protein sources trigger a similar appetite response to animal sources (97). Consequently, the agreement between the present and previous studies that predominately investigated milk-based ONS is therefore not surprising. Moreover, in the present study, we did not control when patients consumed the plant-based ONS, which is noteworthy as timing of ONS consumption may affect appetite and energy intake (62). Patients consumed the plant-based ONS across the day, including with breakfast (13%), lunch (17%), and dinner (13%), but the majority consumed the plant-based ONS between meals (58%). In agreement with previous work (22), this study indicates that ONS consumed between meals seems most accepted and feasible to maintain high compliance and patient appetite/energy intake. Importantly, results from the present study also demonstrated that appetite and energy intake from diet alone was maintained following consumption of a plant-based ONS, no matter the time of day consumed.

Overall, GI tolerance was stable while patients consumed the plant-based ONS and no SAEs were reported, confirming the tolerability and safety of plant-based ONS in patients at risk of DRM. This data concurs with previous work on alternative ONS (2, 18, 98, 99). However, it is important to state that the present study observed increases in mild symptoms of flatulence and burping, and adverse events of mild–moderate abdominal pain, acid reflux and nausea were reported. Though, adverse events were only reported by 3 (13%) patients, and while these patients terminated the intervention period early, they still reported a high mean compliance of 70% (SD 40) with the plant-based ONS. Minor GI symptoms are typically the most common adverse event and have previously been reported following consumption of ONS in a small number of patients at risk of DRM (2, 98, 100); therefore, minor symptoms observed in a small number of patients in this study is consistent with previous studies. In addition, our sub-analysis reported that in patients already prescribed an ONS at baseline, no differences in GI symptoms occurred. This may suggest, although cannot be confirmed in this study, that the new addition of ONS and not the plant-based nature of the ONS *per se*, contributed to the mild increases in flatulence and burping. Nevertheless, most patients (79%) strongly agreed or agreed that the plant-based ONS was well tolerated, confirming that a plant-based ONS is likely tolerated in the majority of patients at risk of DRM.

### Strengths and limitations

The strengths of this study include the novel collection of a wide array of outcomes in real-world, incorporating nutritional outcomes and motivations for plant-based ONS, in a population at risk of DRM following a plant-based ONS. However, while this study makes a valuable contribution to the evidence base for the use of ready-to-drink, plant-based ONS in the management of DRM, it has several limitations that warrant further discussion.

Firstly, the sample size was small and the study design was single arm, lacking a control group. The sample size was based on a power calculation to observe an increase in compliance to ONS of 9% (90% compliance). At the end of intervention, we observed greater compliance to the plant-based ONS (94%), however, prior to the study, it was anticipated that most patients would have already been prescribed an ONS at baseline, but 67% were not. Therefore, only a sub-analysis of *n* = 8 patients was conducted for the primary outcome. Nevertheless, in these patients, while not significant, compliance increased by 26%. The study also mimicked clinical practice, whereby patients newly identified at risk of DRM and requiring intervention were automatically provided dietary advice and prescribed an ONS. The findings of this study of a plant-based ONS are therefore clinically meaningful. Furthermore, although patients served as their own control during the 1-day baseline period, a comparison to a separate control group (e.g., dietary advice alone or a milk-based ONS) would have allowed for a greater interpretation of outcomes. Secondly, there was no standardization at baseline [i.e., prescription of ONS or not and type of ONS (if applicable)], therefore, there was likely heterogeneity between patients on key outcomes. Still, as previously mentioned, we opted for this design to mimic current clinical practice. Thirdly, the study is limited by the short intervention period, and greater clinically applicable findings would likely have been gathered over a longer duration. However, it is important to note that the present study was a pilot study and we observed clinically significant findings in as little as ~26 days. Fourthly, this study investigated the effects of a plant-based ONS alongside dietary advice without accounting for the effects of dietary advice alone. Therefore, the true individual effect of the plant-based ONS is unknown. In clinical practice, however, ONS are routinely prescribed alongside dietary advice (13), hence the present study was designed to follow clinical guidelines. Furthermore, a recent meta-analysis reported that dietary advice alone offers only small effects on patient outcomes for the management of DRM (17), and 54% of patients in this study were following dietary advice alone prior to the study with little to no effect. Consequently, it is likely that the nutritional benefits observed in this study can predominately be attributed to the plant-based ONS. Fifthly, 24 h dietary recalls were used to record patients’ dietary intake and unvalidated questionnaires were used to evaluate GI tolerance and acceptability. Potential reporting bias of 24 h dietary recalls is well documented (101), including under- and/or over-reporting, particularly when intakes are recorded by the patient’s HCP. Although such assessment allows for collection of dietary intake in a community-based study, these drawbacks should be considered when interpreting nutritional intake data in this study. While unvalidated questionnaires were used to assess GI tolerance and acceptability, these questionnaires have been used in previous studies effectively to determine effects of nutritional support (102, 103). Sixthly, confounding variables such as time of day the plant-based ONS was consumed and regular measurement of physical activity levels, which may have impacted the results, were not controlled for, however these variables were recorded and did not change during the intervention period. Nevertheless, as mentioned, this study mimicked clinical practice where patients would consume ONS when most suitable to them. We were also cautious to not place too much burden on patients, many of whom were diagnosed with complex chronic diseases. Finally, as previously mentioned, no measures of lean mass, muscular strength or other functional and clinical outcomes were collected in this study, which would have been valuable data to determine the effectiveness of a plant-based ONS in patients who were likely experiencing sarcopenia and/or cachexia. However, previous studies have shown that ~ ≥ 10–12 weeks is required to determine meaningful changes in such outcomes from oral nutritional intervention (104, 105); therefore, it is unlikely that meaningful changes would have been observed during the intervention timeframe used in this study.

### Clinical implications, applications, and recommendations for future research

The results of this study have important clinical implications and applications for patients at risk of DRM and for HCPs. Primarily, these findings confirm acceptability and feasibility of plant-based nutritional support in the form of a plant-based ONS in patients at risk of DRM. As such, HCPs can now prescribe suitable ready-to-drink plant-based ONS (which are multi-nutrient and nutritionally complete) with evidence-based practice, providing confidence on good compliance, acceptability and GI tolerance, and beneficial effects on body weight and nutritional intake for patients at risk of DRM. In addition, this study highlights that patients may request plant-based nutritional support for a variety of reasons, not only due to veganism or vegetarianism. Therefore, plant-based nutritional support should be offered alongside other forms of nutritional support (e.g., those including animal-derived ingredients) by HCPs to all patients to provide greater variety and patient personalization.

While the present pilot study provides novel preliminary data, additional investigation is needed to further build the evidence base for plant-based nutritional support. Firstly, longitudinal studies (>3 months) with a larger sample size are recommended to confirm the long-term implications. In particular, long-term studies are required on the effects on muscle outcomes and other clinical outcomes such as complications and mortality. Secondly, randomized-controlled trials are necessary to compare plant- to animal-based nutritional support, including investigation of mechanisms of action. Finally, investigation is needed on other applications of plant-based nutritional support, such as enteral tube and infant feeding.

## Conclusion

The results of this single-arm, longitudinal, interventional, multi-center pilot study demonstrate that a ready-to-drink plant-based ONS is highly complied with, tolerated, acceptable and safe, and is effective at increasing total energy, protein and micronutrient intakes, body weight and BMI, and reducing malnutrition risk. Additionally, this study highlights that there are a variety of reasons why patients at risk of DRM may require or prefer a ready-to-drink, plant-based ONS. The major influencing factor that likely explains the high compliance and acceptability, and subsequent nutritional benefits, is the novelty of the plant-based ONS (containing all plant-based ingredients and being suitable for vegan patients), therefore offering a new option and additional variety to patients. Further longer-term investigation is required to ascertain the clinical benefits of using a plant-based ONS in the management of patients at risk of DRM.

## Data availability statement

The original contributions presented in the study are included in the article/Supplementary material, further inquiries can be directed to the corresponding authors.

## Ethics statement

The study involving humans was approved by North West – Greater Manchester West Research Ethics Committee. The study was conducted in accordance with the local legislation and institutional requirements. The participants provided their written informed consent to participate in this study.

## Author contributions

MD: Conceptualization, Data curation, Formal analysis, Investigation, Methodology, Project administration, Validation, Visualization, Writing – original draft, Writing – review & editing. CG: Conceptualization, Data curation, Formal analysis, Investigation, Methodology, Project administration, Validation, Visualization, Writing – original draft, Writing – review & editing. RS: Investigation, Writing – review & editing. ToC: Investigation, Writing – review & editing. HS: Investigation, Writing – review & editing. AV: Investigation, Writing – review & editing. SD: Investigation, Writing – review & editing. NiW: Investigation, Writing – review & editing. JT: Investigation, Writing – review & editing. NM: Investigation, Writing – review & editing. NO: Investigation, Writing – review & editing. JL: Investigation, Writing – review & editing. NaW: Investigation, Writing – review & editing. LiA: Investigation, Writing – review & editing. HO: Investigation, Writing – review & editing. CH: Investigation, Writing – review & editing. MS: Investigation, Writing – review & editing. KGaf: Investigation, Writing – review & editing. KGar: Investigation, Writing – review & editing. SF: Investigation, Writing – review & editing. AS: Investigation, Writing – review & editing. EC: Investigation, Writing – review & editing. SM: Investigation, Writing – review & editing. NB: Investigation, Writing – review & editing. JR: Investigation, Writing – review & editing. JoeA: Investigation, Writing – review & editing. CV: Investigation, Writing – review & editing. TeC: Investigation, Writing – review & editing. CM: Investigation, Writing – review & editing. TT: Investigation, Writing – review & editing. DS: Investigation, Writing – review & editing. JB: Investigation, Writing – review & editing. RM: Investigation, Writing – review & editing. LG: Investigation, Writing – review & editing. KV: Investigation, Writing – review & editing. SR: Investigation, Writing – review & editing. A-MH: Investigation, Writing – review & editing. DM: Investigation, Writing – review & editing. SuB: Investigation, Writing – review & editing. PM: Investigation, Writing – review & editing. SW: Investigation, Writing – review & editing. LB: Investigation, Writing – review & editing. LoA: Investigation, Writing – review & editing. SA-M: Investigation, Writing – review & editing. JoaA: Investigation, Writing – review & editing. SaB: Investigation, Writing – review & editing. RC: Conceptualization, Methodology, Project administration, Supervision, Validation, Visualization, Writing – review & editing. GPH: Conceptualization, Data curation, Funding acquisition, Methodology, Project administration, Resources, Supervision, Validation, Visualization, Writing – review & editing. RJS: Funding acquisition, Project administration, Resources, Supervision, Validation, Writing – review & editing.
