# Supplementary material for: A multi-center prospective study of plant-based nutritional support in adult community-based patients at risk of disease-related malnutrition
Source: Front Nutr. 2023 Nov 10;10:1297624. doi: 10.3389/fnut.2023.1297624 (PMC10667471; doi:10.3389/fnut.2023.1297624)
Supplement: Supplementary file 1 [file Table_1.DOCX]

**Supplementary Table 1. Patients Baseline Characteristics**

| **Patient ID.** | **Sex** | **Age (yrs)** | | **Weight (kg)** | | **BMI (kg/m^2^)** | | **MUST**  **Score** | | **Diagnosis** | | **Indication for ONS at baseline** | | **Indication for**  **Plant-based ONS** | | **No. of days on ONS** | |
| --- | --- | --- | --- | --- | --- | --- | --- | --- | --- | --- | --- | --- | --- | --- | --- | --- | --- |
| 040 | Female | 68 | | 50.0 | | 17.3 | | 2 | | COPD | | Patient had low BMI, ‘MUST’ score of 2, and could not maintain body weight. Dietary advice provided, but ONS required | | Sustainability reasons | | 28 | |
| 657 | Female | 73 | | 45.0 | | 18.0 | | 2 | | Congestive  Heart Failure | | Patient unable to gain weight with current diet following cardiac operation, ‘MUST’ score of 2, felt weak and required ONS to regain body weight | | Cultural/religious reasons | | 28 | |
| 674 | Male | 63 | | 76.0 | | 25.7 | | 2 | | Lung Cancer | | Weight loss >10% in previous few months leading to ‘MUST’ score of 2, poor appetite, provided dietary advice before study but nutritional intake/weight not improved | | Personal preference/variety | | 28 | |
| 171 | Female | 36 | | 45.0 | | 17.5 | | 2 | | Endometriosis | | BMI <18 kg/m^2^, ‘MUST’ score of 2, provided dietary advice and ONS but weight not improved | | Patient wished to reduce animal-derived consumption | | 28 | |
| 257 | Female | 76 | | 33.0 | | 14.5 | | 2 | | Significant weight loss – under investigation for CVD | | ‘MUST’ score of 2 with low BMI and continued to lose weight, dietary advice provided, patient felt weak and required ONS to gain weight | | Sustainability reasons | | 12 | |
| 659 | Female | 74 | | 40.0 | | 15.6 | | 2 | | Total gastrectomy due to stomach cancer | | ‘MUST’ score of 2 with low BMI, patient weak and frail, provided dietary advice but struggled to consume food and had not gained weight | | Personal preference/variety | | 28 | |
| 943 | Female | 63 | | 55.0 | | 22.2 | | 2 | | Rectal Carcinoma | | Significant cancer-related weight loss (>10% in last few months) and ‘MUST’ score of 2, patient had poor appetite | | Cultural/religious reasons | | 28 | |
| 160 | Male | 72 | | 50.0 | | 19.1 | | 2 | | COPD | | ‘MUST’ score of 2, dietary advice provided but unable to maintain weight and already prescribed ONS for treatment of DRM | | Reason not provided | | 28 | |
| 562 | Male | 76 | | 69.0 | | 22.9 | | 0 | | Malignant neoplasm of rectum | | Dietary advice provided but unable to maintain weight, patient had inability to consume food and had reduced appetite, patient already prescribed ONS for treatment of DRM | | Personal preference/variety | | 28 | |
| 401 | Female | 29 | | 47.6 | | 17.6 | | 2 | | Anorexia Nervosa | | Patient had low BMI and ‘MUST’ score of 2, was already prescribed ONS, regularly missed meals and required ONS to increase calorie intake to prevent further weight loss | | Patient wished to reduce animal-derived consumption | | 28 | |
| 642 | Female | 34 | | 49.3 | | 19.3 | | 1 | | Weight loss related to anxiety | | Weight loss, low BMI, unable to gain weight with current vegan diet and required ONS to gain weight | | Patient wanted a vegan ONS | | 28 | |
| 245 | Female | 56 | | 39.6 | | 15.5 | | 2 | | COPD | | Low BMI and ‘MUST’ score of 2, patient unable to maintain required nutritional intake for weight gain with current diet and dietary advice, poor desire to eat food and already prescribed ONS to treat DRM | | Reason not provided | | 21 | |
| 204 | Male | 31 | | 52.0 | | 17.0 | | 2 | | Epilepsy | | Low BMI and ‘MUST’ score of 2, already prescribed ONS, weight loss continuing due to seizures, dietitian-led dietary advice not leading to weight gain | | Personal preference/variety | | 28 | |
| 543 | Female | 74 | | 44.0 | | 17.9 | | 2 | | Ischemic Heart Disease | | Low BMI with ‘MUST’ score of 2, reduced appetite and unable to gain weight with diet | | Sustainability reasons | | 28 | |
| 054 | Female | 81 | | 47.0 | | 22.7 | | 2 | | CVD | | Weight loss >10% in last few months, ‘MUST’ score of 2, patient had poor appetite and no desire to eat food, frail and unable to gain weight with diet | | Cultural/religious reasons | | 16 | |
| 097 | Male | 42 | | 48.0 | | 17.5 | | 2 | | Hypertensive Heart Disease | | Low BMI and ‘MUST’ score of 2, poor appetite and no desire to eat food, unable to gain weight with diet | | Personal preference/variety | | 28 | |
| 822 | Female | 83 | | 40.0 | | 17.30 | | 2 | | COPD | | Low BMI, not able to meet nutritional requirements with diet alone. Required ONS to gain weight | | Personal preference/variety | | 28 | |
| 666 | Female | 46 | | 46.0 | | 18.5 | | 1 | | Anorexia Nervosa | | Eating disorder and psychosis with poor desire to eat food | | No specific reason | | 28 | |
| 524 | Female | 64 | | 41.6 | | 18.0 | | 2 | | Anxiety - unable to maintain weight | | Low BMI. Required ONS to increase weight and strength | | No specific reason | | 28 | |
| 756 | Female | 84 | | 66.0 | | 29.2 | | 2 | | Rheumatoid Arthritis | | >13.6% weight loss, difficulty to eat food due to arthritis. Felt weak | | Cultural/religious reasons | | 28 | |
| 754 | Female | 60 | 50.0 | | 18.4 | | 2 | | Lung Cancer | | Low BMI with ‘MUST’ score of 2, reduced appetite and unable to gain weight with diet | | Cultural/religious reasons | | 7 | |  |
| 933 | Female | 36 | 60.6 | | 18.8 | | 1 | | Loeys-Dietz syndrome with associated progressive aortic and peripheral arterial aneurysmal disease | | Low BMI and unable to get sufficient energy intake just from diet | | No specific reason | | 28 | |  |
| 872 | Female | 32 | 53.6 | | 17.8 | | 2 | | Subclinical Hypothyroidism | | Low BMI with ‘MUST’ score of 2, reduced appetite and unable to gain weight with diet | | No specific reason | | 28 | |  |
| 421 | Male | 51 | 50.0 | | 17.30 | | 2 | | Ulcerative colitis | | Low BMI with ‘MUST’ score of 2, unable to gain weight with food only | | Health reasons | | 28 | |  |
